# Carbon-ion radiotherapy for marginal lymph node recurrences of cervical cancer after definitive radiotherapy: a case report

**DOI:** 10.1186/1748-717X-8-79

**Published:** 2013-04-05

**Authors:** Tomoaki Tamaki, Tatsuya Ohno, Hiroki Kiyohara, Shin-ei Noda, Yu Ohkubo, Ken Ando, Masaru Wakatsuki, Shingo Kato, Tadashi Kamada, Takashi Nakano

**Affiliations:** 1 Department of Radiation Oncology, Gunma University Graduate School of Medicine, 3-39-22 Showa-machi, Maebashi-shi, Gunma, 371-8511, Japan; 2 Research Center for Charged Particle Therapy, National Institute of Radiological Sciences, 4-9-1 Anagawa, Inage-ku, Chiba-shi, Chiba, 263-8555, Japan; 3 Saitama Medical University International Medical Center, 1397-1 Yamane, Hidaka-shi, Saitama, 350-1298, Japan

**Keywords:** Carbon-ion radiotherapy, Marginal recurrences, Cervical cancers

## Abstract

Recurrences of cervical cancer after definitive radiotherapy often occur at common iliac or para-aortic lymph nodes as marginal lymph node recurrences. Patients with these recurrences have a chance of long-term survival by optimal re-treatment with radiotherapy. However, the re-irradiation often overlaps the initial and the secondary radiotherapy fields and can result in increased normal tissue toxicities in the bowels or the stomach. Carbon-ion radiotherapy, a form of particle beam radiotherapy using accelerated carbon ions, offers more conformal and sharp dose distribution than X-ray radiotherapy. Therefore, this approach enables the delivery of high radiation doses to the target while sparing its surrounding normal tissues. Marginal lymph node recurrences in common iliac lymph nodes after radiotherapy were treated successfully by carbon-ion radiotherapy in two patients. These two patients were initially treated with a combination of external beam radiotherapy and intracavitary and interstitial brachytherapy. However, the diseases recurred in the lymph nodes near the border of the initial radiotherapy fields after 22 months and 23 months. Because re-irradiation with X-ray radiotherapy may deliver high doses to a section of the bowels, carbon-ion radiotherapy was selected to treat the lymph node recurrences. A total dose of 48 Gy (RBE) in 12 fractions over 3 weeks was given to the lymph node recurrences, and the tumors disappeared completely with no severe acute toxicities. The two patients showed no evidence of disease for 75 months and 63 months after the initial radiotherapy and for 50 months and 37 months after the carbon-ion radiotherapy, respectively. No severe late adverse effects are observed in these patients. The two presented cases suggest that the highly conformal dose distribution of carbon-ion radiotherapy may be beneficial in the treatment of marginal lymph node recurrences after radiotherapy. In addition, the higher biological effect of carbon-ion radiotherapy and its superior dose distribution may provide more effective tumor control in treatment for re-irradiation of the marginal recurrences in radiation resistant tumors other than cervical cancer.

## Background

Metastases in common iliac or para-aortic lymph nodes (LN) often occur after definitive radiotherapy (RT) for cervical cancer [1]. RT for these lymph node metastases provides a chance of long-term survival in these patients [2]. However, if the recurrences occur very close to the edge of the initially treated field after RT, there is a concern that an overlap of the initial and the second radiation fields could yield a high cumulative dose and possibly result in severe toxicities in normal tissues such as the bowel. Carbon-ion RT, a form of particle beam RT using accelerated carbon ions, offers more conformal radiation dose distribution than X-ray RT and thus enables the delivery of high radiation doses to the tumor while sparing the surrounding normal tissues [3,4]. Carbon-ion RT has shown excellent local control for X-ray resistant diseases such as recurrent rectal carcinoma, skull-base tumors, uveal melanoma, non-squamous cell carcinomas of the head and neck, and sarcomas. In these tumors, high radiation doses could be delivered to the clinical targets by carbon-ion RT without causing severe complications in the surrounding normal organs [3,4]. In this article, we report two cases of recurrent cervical cancer in the LN marginal to the RT fields utilized in the initial treatment. Definitive carbon-ion RT was applied successfully to the marginal LN recurrences of these patients without further recurrences or severe complications.

## Case presentation

### Patient 1

**Figure 1:**
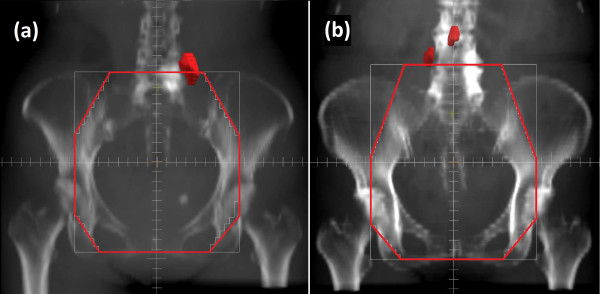
**Location of the lymph node recurrences in relation to the radiation field of the initial radiotherapy.** The initial radiation fields were reconstructed with red lines, while the recurred lymph nodes were contoured as red solids. Lymph nodes recurred at the edge of the initial radiation field both in (**a**) Patient 1 and (**b**) Patient 2.

**Figure 2:**
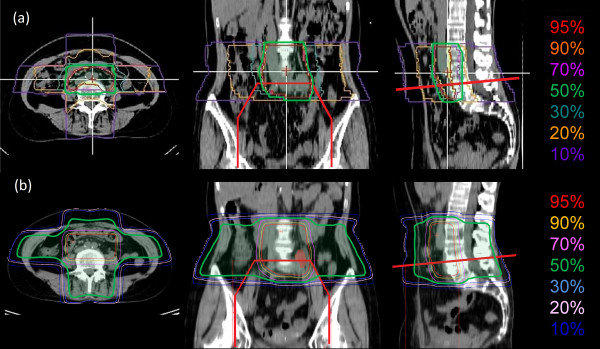
**Comparison of carbon-ion radiotherapy (RT) and X-ray RT dose distributions in Patient 1.** (**a**) Dose distribution of the carbon-ion RT applied in Patient 1 for the lymph node (LN) recurrence. Carbon-ion RT of 48 Gy (RBE)/12 fractions/3 weeks was given to the left common iliac LN and the lower para-aortic LN region at the National Institute of Radiological Sciences, Chiba, Japan. The isodose lines are shown by percentage with respect to the total dose of 48 Gy (RBE). (**b**) Simulated dose distribution of X-ray (RT) using orthogonal 4 fields. The anterior-posterior field of the initial RT is shown with thick red lines. The 50% isodose lines are highlighted in green.

**Figure 3:**
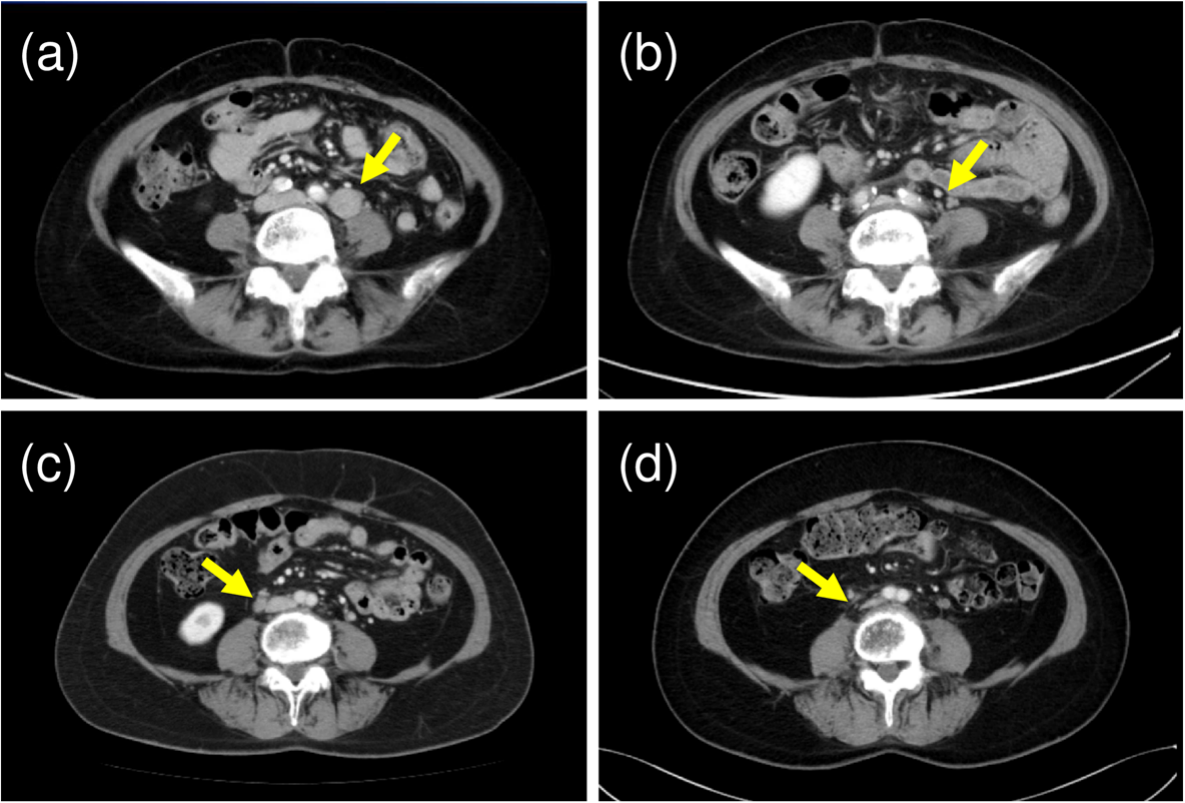
**CT images of the recurred lymph nodes (LNs) at the time of recurrence ((a) Patient 1, (c) Patient 2)) and 3 years after carbon-ion radiotherapy ((b) Patient 1, (d) Patient 2).** The recurrent LNs have disappeared after the therapy in both patients.

A 55-year-old female presented with abnormal vaginal bleeding and was diagnosed with FIGO Stage IIA (T2aN0M0, UICC 6th edition) squamous cell carcinoma of the uterine cervix. The patient had an elevated value of serum squamous cell carcinoma antigen (SCC), 5.3 ng/ml (normal value: 0–1.5 ng/ml). The patient was treated with external beam RT (EBRT) of 50 Gy in 25 fractions to the pelvis using conventional X-rays (consisting of 26 Gy of whole pelvis fields and the last 24 Gy of center shielding pelvis fields) and 4 fractions of high-dose-rate (HDR) intracavitary brachytherapy (BT) with the total dose of 24 Gy given to point A. There were no acute adverse effects greater than Grade 3. However, there were Grade 2 upper gastrointestinal symptoms and Grade 1 neutropenia. The tumor completely disappeared, and the serum SCC levels returned to normal after therapy. Therefore, the patient received no further treatment. Twenty-three months later, the patient developed bilateral edema in the legs. A contrast-enhanced computed tomography (CT) study showed an enlarged left common iliac LN with a diameter of 2 cm situated at the edge of the previous whole pelvis irradiation field (Figure 1(a)). Based on an elevated uptake of 18F-fluorodeoxy glucose (FDG) in the positron-emission tomography (PET)/CT study (maximum SUV 6.1) and an elevated value of serum SCC (4.0 ng/ml), the enlarged LN was clinically diagnosed as a metastasis of cervical cancer. To avoid normal tissue complications from re-irradiation, carbon-ion RT was selected as the treatment modality. Carbon-ion RT (48 Gy (RBE) in 12 fractions over 3 weeks) was administered to the left common iliac LN and the lower para-aortic LN (PALN) region (Figure 2a). The patient experienced no serious adverse effects during the therapy, and no further therapy was administered. The serum SCC level returned to normal levels (<1.0 ng/ml) after the carbon-ion RT treatment. The patient showed no evidence of disease for 75 months after the initial RT and for 50 months after the carbon-ion RT of the LN recurrence (Figure 3). There were no serious adverse effects beside the leg edema that was present prior to treatment.

### Patient 2

A 52-year-old female patient presented with abnormal vaginal bleeding and was diagnosed with squamous cell carcinoma of uterine cervix. She was referred to our department for concurrent chemoradiotherapy. A colonoscopy confirmed the invasion of the tumor into the rectal mucosa, and the patient was staged to be FIGO Stage IVA (T4N0M0, UICC 6th edition). The patient was treated with EBRT of 50 Gy in 25 fractions to the pelvis using conventional X-rays (consisting of 30 Gy of whole pelvis fields and 20 Gy of center shielding pelvis fields) and 5 fractions of HDR BT (2 fractions of intracavitary BT with 6 Gy prescribed to the point A and 3 fractions of image-guided interstitial BT with 6 Gy prescribed to the gross tumor). The patient also received 4 courses of weekly cisplatin at a dose of 40 mg/m^2^. Although Grade 2 upper gastrointestinal symptoms and leukopenia were observed during the treatment, the tumor disappeared completely. Twenty-two months later, a follow-up contrast-enhanced CT and FDG-PET/CT showed an enlarged PALN (10 mm in diameter, max SUV 3.10) and an enlarged right common iliac LN (10 mm in diameter, max SUV 2.81) at the edge of the whole pelvis irradiation field utilized in the initial treatment (Figure 1(b)). These lymph nodes were clinically diagnosed as metastases of cervical cancer. Similar to the previous patient, carbon-ion RT with the dose of 48 Gy (RBE) in 12 fractions over 3 weeks was administered to the common iliac LN and lower PALN regions. The patient was discharged with no apparent acute adverse effects. No further therapy was administered, and the patient showed no evidence of further recurrences for 63 months after the initial RT and for 37 months after the carbon-ion RT for the recurred LN (Figure 3). The patient experienced no late toxicities from the treatment.

## Discussion

Metastases in the common iliac LN and PALN are often observed after definitive RT for cervical cancers [1]. A retrospective study of 1894 patients who received definitive RT for cervical cancer at the M.D. Anderson Cancer Center has shown that the most common site of regional recurrence in patients with centrally controlled disease was the cranial margin of the initial RT fields. In the study, 198 patients (10.5%) had regional recurrences with no central recurrence. Of these patients, 75 patients (4.0%) were confirmed with diagnostic images to have only marginal recurrences. Most of the recurrences were located in the superior margin of the initial RT fields (73 patients) [1]. In other prospective clinical studies, the PALN recurrences were observed in 7% of patients treated with chemoradiotherapy in the Radiation Therapy Oncology Group 90–01 trial [5]. Additionally, PALN recurrences were found in 15% of patients in the Japanese Gynecologic Oncology Group 1066 trial that studied patients with more advanced diseases of FIGO Stage III-IV [6].

The 5-year overall survival of patients with solitary LN metastases that are marginal to the initial radiation fields was 13% in the M.D. Anderson Cancer Center study [1]. In contrast, it is reported that the 3-year and 5-year overall survival of patients who received X-ray RT for solitary PALN recurrence of cervical cancer were 50% and 31%, respectively [2]. It was also found that the delivery of radiation doses above 50 Gy was a significantly favorable factor for the survival of these patients [2]. These results indicate that patients with solitary recurrence in PALN or in the common iliac LN would be candidates for definitive RT and could achieve long-term survival if an adequate amount of radiation dose can be safely delivered to the LN recurrence.

**Figure 4:**
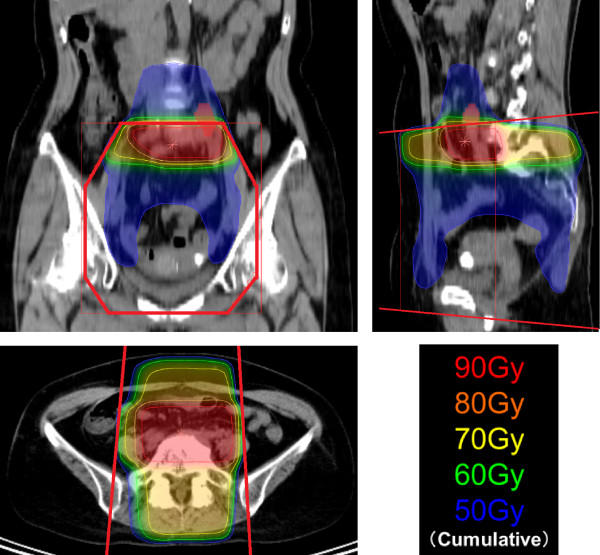
**Simulated diagram of cumulative dose distribution in Patient 1.** The dose distribution was simulated in a case if the re-irradiation of 50 Gy were to be administered by 3D conformal X-ray radiotherapy using an orthogonal 4-field technique. The cumulative radiation dose to the bowels is greater than 70 Gy because of the overlap of the initial and re-treatment radiation fields. The red lines indicate the anterior-posterior field of the initial radiotherapy.

RT to the abdomen requires careful attention to prevent adverse effects in the gastrointestinal tract, especially in the stomach and small bowels. The radiation dose-volume analyses of normal tissue toxicities recommend the volume of small bowels receiving doses above 45 Gy should be minimized [7]. To prevent the incidence of severe normal tissue toxicities in cases of re-irradiation, physicians should avoid or minimize the volume receiving a high cumulative dose due to the overlap of the initial and re-treatment irradiation fields. In our simulation study of re-irradiation using conventional X-ray treatment by 3D conformal RT, a strip of small bowel is exposed to a cumulative irradiation dose above 70 Gy (Figure 4) due to the overlap of radiation fields. Thus, re-irradiation with X-ray RT could result in a significant risk of complications in small bowels.

Alternatively, carbon-ion RT has been shown to have higher anti-tumor effects and superior dose distributions compared to conventional X-ray RT [3,4,8]. The superior dose distribution of carbon ion beams results from the ability of accelerated carbon ions to release a maximal amount of energy at the end of the track as a so-called Bragg peak. This energy release allows a smaller amount of lateral scattering (penumbra) due to the greater mass of carbon nuclei [3,4]. Due to the differences of treatment planning systems between the carbon-ion RT and X-ray RT, there is a limitation in the graphical presentation of cumulative dose distributions of these patients. However, while the overlap of the treatment fields could not be completely avoided, the application of carbon-ion RT could significantly reduce the volume of small bowel receiving high doses (Figure 2). As a result, the patients did not have severe late toxicities. A possible uncertainty in carbon-ion RT applied to abdomen is that its dose distribution can be affected by the excessive fluctuation of bowel gas in the beam track. In order to minimize such uncertainty, the presence of bowel gas is checked with daily fluoroscopic images used for patient set-up. The two patients will continue to be followed carefully and the incidence of late toxicities will be monitored. These cases support previous reports that suggested patients with marginal LN recurrences after definitive RT of cervical cancers can possibly achieve long-term survival if enough radiation is delivered to the target. Carbon-ion RT is one of the modalities that may enable re-irradiation for these patients in a safer manner.

## Conclusions

In summary, in two patients with solitary marginal LN recurrences after definitive RT, carbon-ion RT was successfully applied to the lesions while minimizing high doses to the bowels. The patients are disease free and do not have late toxicities. Carbon-ion RT may be an effective treatment option for re-irradiation in the marginal recurrences of cervical cancer and other malignant tumors.

## Consent

Written informed consent was obtained from the patients for publication in this case report and any accompanying images. A copy of the written consent is available for review by the Editor-in-Chief of this journal.

## Abbreviations

RT: Radiotherapy; LN: Lymph nodes; FIGO: International federation of gynecology and obstetrics; UICC: Union for international cancer control; EBRT: External beam radiotherapy; HDR: High-dose-rate; BT: Brachytherapy; CT: Computed tomography; FDG: ^18^F-fluorodeoxy glucose; PET: Positron emission tomography; RBE: Relative biological effectiveness; Gy: Gray; PALN: Para-aortic lymph nodes.

## Competing interests

The authors declare that they have no competing interests.

## Authors’ contributions

TT analyzed the treatments and drafted the manuscript. TO analyzed the treatment and contributed to the final draft of the manuscript. HK, YO, KA, MW, SK, and TK planned the treatment and contributed to the final draft of the manuscript. SN analyzed the treatment and contributed to the manuscript. TN analyzed the treatment and contributed to the final draft of the manuscript. All authors read and approved the final manuscript.
